# Regional Technique Provides Complete Surgical Anesthesia for Above-the-Knee Amputation: A Viable Alternative to General Endotracheal Anesthesia in a Time of COVID-19

**DOI:** 10.7759/cureus.25364

**Published:** 2022-05-26

**Authors:** Mary Kay Kujak, Lauren H Pomerantz, Michelle Petrovic

**Affiliations:** 1 Anesthesiology, University of Central Florida College of Medicine, Orlando, USA; 2 Medicine, University of Central Florida College of Medicine, Orlando, USA; 3 Anesthesiology, Orlando Veterans Affairs (VA) Medical Center, Orlando, USA

**Keywords:** regional anesthesiology, nerve block management, indications for intubation, covid-19, peripheral nerve block

## Abstract

In the initial phase of the coronavirus disease 2019 (COVID-19) pandemic, laboratory test shortages made it impossible to definitively identify patients with active COVID-19 infection or asymptomatic carriers. Without diagnostic certainty, it was imperative to proceed with caution when performing aerosol-generating procedures; this meant that anesthesiologists needed to make conscious decisions to avoid airway manipulation for procedures when it was not completely necessary. This case report describes a regional anesthetic technique that was used as the primary anesthetic for an urgent above-the-knee amputation in a patient with a history of respiratory issue of unknown etiology.

## Introduction

The coronavirus disease 2019 (COVID-19) pandemic was classified into a highly contagious respiratory virus that yielded symptoms of severe respiratory distress, shortness of breath, fever, dry cough, sore throat, aches, and pains. First diagnosed in December 2019, COVID-19 was difficult to identify in both symptomatic and asymptomatic patients due to test shortages. In the absence of diagnostic certainty, it was imperative to proceed with caution when it came to performing aerosol-generating procedures. This meant making conscious decisions to entirely avoid airway manipulations.

Amid the COVID-19 pandemic, anesthesiologists were required to assess the efficacy of previously studied methods of anesthetic management for major surgical procedures, such as lower extremity amputations, to determine options other than general anesthesia. One such study was a retrospective descriptive study that sought to evaluate peripheral nerve block (PNB) success in high-risk patients as the sole anesthetic for an above-the-knee amputation (AKA). This report defined high risk as patients who were given an American Society of Anesthesiologists Physical Status (ASA PS) score of IV or greater and defined success as not needing to convert to a general or spinal anesthetics [1]. Of the 57 patients in this study, 91% successfully had an AKA with PNB, but 95% of the patients required additional sedation and analgesia during the procedure in addition to PNB [1]. There were three different blocks used in this study: femoral, obturator, and sciatic (FOS), FOS plus lateral femoral cutaneous (FOSL), and femoral and sciatic (FS). FOSL blocks required less additional sedation analgesia intraoperatively than FOS and FS blocks [1]. Ultimately, PNB strategy had an overall benefit on the mortality rate in ASA IV patients, with 30-day mortality having a decrease from the national standard of 23.2% to only 12.3% [1]. Therefore, PNB was found not only to be a viable option for sole anesthetic in severely ill patients undergoing AKA but also to provide mortality benefit.

Pre-COVID-19, our facility routinely used general endotracheal anesthesia (GETA) for AKA and had never used a regional anesthesia technique before for this type of case. Unlike for below-the-knee amputations (BKA), PNBs were not normally used for an AKA as the primary anesthetic due to the extensive anatomical coverage needed. Our case describes a PNB technique that was successfully used as the primary anesthetic for an AKA.

Written Health Insurance Portability and Accountability Act (HIPAA) authorization and consent was obtained from the patient’s next of kin to publish this report.

## Case presentation

A 72-year-old male with a past medical history of coronary artery disease, hypertension, peripheral vascular disease, moderate pulmonary hypertension, end-stage renal disease on hemodialysis, cardiomyopathy with an ejection fraction of 45-50%, and insulin-dependent diabetes mellitus presented with an urgent need for left-sided AKA due to severe vascular disease causing vascular compromise of the limb. The patient’s past surgical history was significant for left Cimino fistula, femoral-popliteal arterial bypass, and partial hallux amputation. Of note, the patient had a respiratory issue of unconfirmed etiology in early February of 2020; while unconfirmed, based on symptoms experienced and contact tracing, this was likely COVID-19.

When he arrived at the preoperative bay, he was wearing a commercial N95 mask, was afebrile, and was asymptomatic from a respiratory standpoint. The case occurred in late March 2020 when there were limited N95 masks available and conflicting local and national protocols existed regarding their distribution and use. In the preoperative bay before the case, tensions were high and often pitted providers from different specialties against each other. For example, non-operating room (OR) personnel questioned whether OR personnel should be allowed to wear N95 masks in the care of non-COVID-19 patients during aerosol-generating procedures. Amidst this uncertainty, the anesthesiologist, who was highly trained in regional anesthesia, decided to avoid aerosol-generating intubation by performing the AKA entirely under PNB. The anesthesiologist can also consider implementing neuraxial anesthesia in the form of epidural or spinal. In this case, regional was chosen as it has been proven to provide greater hemodynamic stability and required less use of vasopressors or cardiac agents [1]. It has also been found that it is "unsafe to perform neuraxial blocks on high-risk patients with deranged coagulation secondary to sepsis or the use of anticoagulant/antiplatelet medications" [1].

Four total ultrasound-guided blocks were performed: subgluteal sciatic, femoral, obturator, and lateral femoral cutaneous. For the subgluteal sciatic block, the injection was placed between the adductor magnus muscle and biceps femoris muscle. For the femoral block, the injection site was located below the inguinal crease and immediately lateral to the femoral artery in order to anesthetize the anterior and medial thigh down to and including the knee and a variable amount of skin on the medial leg and foot. For the obturator block, the ultrasound aided in guiding the anesthesiologist to inject medication between the pectineus and adductor brevis in order to anesthetize the cutaneous aspect of the medial thigh. For the lateral cutaneous block, the injection site was located between the tensor fasciae latae muscle (TFLM) and the sartorius muscle (SaM) by locating the anterior superior iliac spine in order to anesthetize the anterolateral aspect of the thigh. Ultimately, these four blocks allowed for the achievement of both motor and sensory blockage below the knee. All dermatomes were effectively blocked. A solution of 1:1 bupivacaine 0.5% and lidocaine 2% was used: 20 cc was given in the subgluteal sciatic block, 10 cc in the femoral block, and 5 cc in both the obturator and the lateral femoral cutaneous blocks. A total of 50 cc of local anesthetic was given. The patient’s preblock pain score was 10/10, and after 20 minutes his pain score was 0/10. No redosing of local anesthetic was needed.

No airway manipulation occurred during surgery as the patient’s oxygen mask was placed on top of his home N95 mask. The four blocks provided complete surgical anesthesia, and intraoperatively he was only given 100 mcg of fentanyl, 2 mg of midazolam, and low-dose propofol ranging from 25 to 30 mcg/kg/min. The procedure was successful with no complications. However, the patient died 30 days later from acute hypoxemic respiratory failure secondary to COVID-19 pneumonia.

## Discussion

Major lower extremity amputation is a high-risk surgery that poses a significant mortality risk for both BKA and AKA. One retrospective study looking at the national patterns of mortality after major lower extremity amputation in Medicare patients >65 years of age with significant peripheral arterial disease (PAD) found a mortality rate of 13.5% at 30 days, 48.3% at one year, and 70.9% at three years [2]. The hazard ratio (HR) in the BKA group was 1.29, with a 95% confidence interval (CI) of 1.29-1.29 [2]. An AKA had a statistically higher hazard of death when compared to BKA or more distal amputations with an HR of 1.31 and 95% CI of 1.25-1.36 [2]. Another retrospective study evaluating the perioperative and long-term morbidity and mortality of AKA versus BKA reported that AKA surgeries should be triaged as high risk, while BKA surgeries are intermediate risk because cardiac event rate was higher in AKA, and AKA revealed higher mortality [3].

The increased mortality rate in AKA has prompted further investigation into whether the choice of anesthetic contributes to higher mortality and perioperative complications. Upon review, the literature pertaining to this question is mixed. One retrospective cohort using propensity-matched groups of patients undergoing AKA found no difference in the primary outcome of 30-day mortality or secondary outcome of cardiac, pulmonary, infectious, or bleeding complications, or length of stay in patients who used general anesthesia versus regional anesthesia [4]. A different study looking at patients who underwent a major lower extremity amputation found that of the 3,260 patients identified, 2,558 received general anesthesia and 702 received regional anesthesia, while 59% of patients in this study received an AKA and 41% received BKA [5]. The study found no difference between the 30-day mortality rate, postoperative myocardial infarction, cardiac arrest, pulmonary complications, stroke, urinary tract infections, wound complications, and length of stay in groups undergoing general anesthesia versus regional anesthesia [5]. However, a retrospective study from 2002 to 2011 identified patients undergoing major lower extremity amputation and found that there were differences in perioperative complications between types of anesthesia [6]. Patients undergoing regional anesthesia had a lower incidence of postoperative pulmonary complications compared to those undergoing general anesthesia [6]. Length of stay in the intensive care unit and in the hospital was shorter in the regional anesthesia group [6].

The pandemic created a healthcare stress like none other in recent history. It caught most healthcare providers and hospitals unprepared and undersupplied. One of the biggest issues in the early stages was our incomplete understanding of its transmission. There was much debate about whether transmission occurred only by fomite and droplets or whether it could also be aerosolized. The contagiousness of infection (known as R0) can be defined as the average number of people who will contract a disease from one person who is contagious. The R0 for COVID-19 has been shown to be 1.5-3 [7]. However, this number drastically increases within confined spaces to an R0 ranging from 5 to 14 [7]. Therefore, adequate access to and use of personal protective equipment (PPE) was paramount for healthcare worker safety. In the early weeks of the pandemic, PPE options were limited for many healthcare workers across the country.

Furthermore, there was little knowledge about asymptomatic carrier states and their role in the spread of COVID-19. This incomplete understanding combined with the initial absence and shortage of COVID-19 tests created a system where it was a luxury to have a PCR-positive diagnosis whether the patient was asymptomatic or symptomatic. Without protocols in place for the management of suspected, active, prior positive, and asymptomatic COVID-19 patients, there were clinical disagreements between healthcare personnel, especially those from different specialties. Frontline workers had the impossible task of balancing saving scarce PPE resources while simultaneously being proactive in their use of PPE for protection from an unfamiliar virus.

## Conclusions

Since our patient had a history of an unknown respiratory infection amidst a global pandemic, precautions were taken to eliminate airway manipulation unless necessary. This case is important because it is an example of thinking critically to discover creative solutions without a clear path forward. Our team avoided aerosol-generating procedures to protect everyone in the OR while not compromising anything in terms of patient care, comfort, and safety. While this may not be the typical anesthetic approach to an AKA, this four-block technique proved to be successful and is therefore a viable alternative for future patients with high consequence airway infections.
